# Small-Conductance Ca^2+^-Activated K^+^ Channels and Their Regulation of Excitability of Autonomic Neurons

**DOI:** 10.1007/s12264-026-01715-1

**Published:** 2026-09-18

**Authors:** Renjun Wang, Zhengwei Li, Andrew D. Chapp, Min Lin, Dong-Shu Du, Zhiying Shan, Zixi Jack Cheng, Qing-Hui Chen

**Affiliations:** 1 Department of Biotechnology, School of Life Science, Jilin Normal University, Siping 136000, China; 2 Department of Kinesiology and Integrative Physiology, Michigan Technological University, Houghton, MI 49931-1200, USA; 3 Department of Neuroscience, Evelyn F. and William L. McKnight Brain Institute, University of Florida, Gainesville, FL 32611, USA; 4 School of Life Sciences, Shanghai University, Shanghai 200444, China; 5 Biomolecular Science Center, College of Medicine, Burnett School of Biomedical Sciences, University of Central Florida, Orlando, FL 32816, USA

**Keywords:** Hypothalamic paraventricular nucleus, Nucleus ambiguus, SK channels, Hypertension, Neonatal mice of diabetic mothers, Autonomic nervous system

## Abstract

Dyshomeostasis of the autonomic nervous system (ANS) drives chronic illnesses including hypertension, heart failure, diabetes mellitus, metabolic syndrome, Parkinson’s disease, and traumatic brain injury. Abnormal neuronal excitability within central nervous system (CNS) nuclei is a major cause of ANS dysfunction. Small-conductance calcium-activated potassium (SK) channels govern excitability in central autonomic nuclei, sustaining neuronal excitation-inhibition balance *via* medium afterhyperpolarization potentials (mAHP)-mediated negative feedback. This review describes SK channels’ molecular structure and electrophysiology, maps central sympathetic-parasympathetic neuroanatomical pathways, and highlights pathological impacts of the dysfunction of SK channels among the hypothalamic paraventricular nucleus (PVN) and nucleus ambiguus (NA). It also summarizes SK channels-centered regulatory approaches: epigenetics (DNA methylation, miRNA modulation), post-translational modification (phosphorylation, ubiquitination), protein interaction, metabolites, and synthetic exogenous agonists and antagonists. These results offer a theoretical basis and candidate targets to decode the neurogenic hypertension and diabetes mechanisms and facilitate bench-to-clinic translation.

## Introduction

As a branch of the peripheral nervous system, the autonomic nervous system (ANS) regulates the physiological activities of multiple systems through the dynamic balance between the sympathetic and parasympathetic nerves, including cardiovascular system homeostasis [1–3], metabolic processes [4, 5], and digestive system functions [6, 7]. Its balanced state serves as a critical foundation for maintaining organismal homeostasis. When factors such as chronic stress, metabolic disorders, and nerve injury disrupt this balance, leading to excessive sympathetic activation or parasympathetic inhibition, they will directly induce multisystem physiological dysfunction and further act as an important pathological mechanism underlying the occurrence and progression of various chronic diseases [8]. In the existing literature, a large body of research has confirmed that ANS imbalance is closely associated with hypertension (HTN) [9–15], heart failure [16–20], diabetes mellitus (DM) [21–24], metabolic syndrome [25, 26], Parkinson’s disease [27–29] and traumatic brain injury [30–33]. This association acts as an early warning sign for diseases and also affects disease prognosis by exacerbating pathological damage. Therefore, clarifying the underlying mechanisms of this correlation, in particular the regulatory mechanisms of the ANS, bears significant theoretical significance and clinical value for the early identification, intervention, and optimization of therapeutic strategies for relevant diseases.

Notably, the balance of the ANS depends on the precise integration of the central nervous system (CNS) [8, 34, 35]. Both sympathetic and parasympathetic activities are governed by a regulatory network composed of circumventricular organs (CVO). This anatomical basis directly indicates that the functional imbalance of the central regulatory network constitutes the fundamental cause of dysfunction of the ANS [36–38]. When the excitatory homeostasis of neurons in these central nuclei is disrupted, it directly alters the output activity of the sympathetic and parasympathetic nerves *via* downstream neural pathways, ultimately leading to peripheral ANS imbalance [39, 40]. Furthermore, the stability of neuronal excitability in these central nuclei is highly dependent on the functional integrity of ion channels [41]. Currently, a substantial body of research has verified that the dysregulation of various central ion channels constitutes a crucial pathological factor for inducing ANS dysfunction. Involved ion channels include voltage-gated sodium channels [42, 43], voltage-gated potassium channels [44, 45], transient receptor potential channels [46–48], inwardly rectifying potassium channels [49], and calcium-activated potassium channels [50, 51].

Among these ion channels associated with ANS dysfunction, the small-conductance calcium-activated potassium (SK) channels, a subtype of calcium-activated potassium channels, have been the research focus of our team. We have preliminarily confirmed that SK channels are closely correlated with the regulation of neuronal excitability in presympathetic nuclei and the pathogenesis of related diseases. On this basis, the present review will systematically elaborate on the physiological functions, pathological mechanisms, and potential intervention strategies of SK channels in the central regulation of the ANS, in combination with the central anatomical pathways of sympathetic and parasympathetic nerves. It aims to build a theoretical framework for elucidating the molecular mechanisms underlying the central regulation of the ANS and promoting the translation of basic research findings into clinical applications for related diseases.

## Network of Autonomic Nervous System: Anatomical Pathways and Functional Regulation

### Sympathetic Nervous System: Central Origins, Pathways, and Peripheral Target Innervation

The functional regulation of diverse peripheral target organs by the SNS relies on thoracolumbar-originated preganglionic neural pathways that connect specific central autonomic nuclei (e.g., subfornical organ (SFO), organum vasculosum of the lamina terminalis (OVLT), paraventricular nucleus (PVN)) to postganglionic neurons and effector organs or tissues (Fig. 1). The SNS mediates diffuse, system-wide sympathetic outflow to coordinate multi-organ responses. Accumulating evidence has demonstrated that specific molecular mechanisms (e.g., glutamatergic signaling in SFO-PVN pathways, adrenergic receptor diversity in peripheral targets) and physiological roles (e.g., stress-induced cardiovascular activation) underlying its regulatory functions have been delineated.

#### Subfornical Organ and Organum Vasculosum of the Lamina Terminalis

As central interfaces for peripheral signal perception, the SFO and OVLT regulate sympathetic activity *via* synergistic modulation of the PVN. In anesthetized rats, optogenetic activation of glutamatergic neurons in the SFO elevates blood pressure *via* the sympathetic nerve only when the PVN is activated, and this effect is independent of vasopressin. These findings clarify the blood pressure-regulating role of the SFO-PVN pathway [52]. Hypernatremia is able to activate the OVLT glutamatergic neurons *via* epithelial sodium channels (ENaC, also referred to as Na_x_ channels), which can directly project to the parvocellular division of the PVN. In turn, PVN neurons transmit signals to sympathetic preganglionic neurons either directly or by recruiting C1 bulbospinal neurons in the rostral ventrolateral medulla (RVLM). This ultimately leads to an elevation in sympathetic tone and an increase in blood pressure [53].

#### Paraventricular Nucleus of the Hypothalamus

As a principal integration center for sympathetic regulation, the PVN innervates corresponding target organs *via* distinct branched pathways [54]. On the one hand, neurons expressing melanocortin-4 receptor (MC4R) in the PVN project to sympathetic preganglionic neurons in the intermediolateral column (IML) of the thoracic spinal cord, and innervate the kidneys through sympathetic efferent nerves, forming the PVN-IML-kidney pathway [55]. On the other hand, when PVN glutamatergic neurons projecting to the RVLM are activated, they stimulate C1 neurons within the RVLM, thereby enhancing renal sympathetic nerve activity and elevating arterial blood pressure in rats [56].

#### Central Nucleus of the Amygdala

The central nucleus of the amygdala (CeA) serves as a critical initiating nucleus for sympathetic activity. In rats, microinjection of ethanol and its metabolite acetic acid into the CeA can activate NMDA receptors in CeA neurons projecting to the RVLM, thereby triggering sympathetic excitation and an elevation in blood pressure; Moreover, inhibition of RVLM neuronal activity can attenuate this response [57]. In addition, brain slice patch-clamp techniques have further confirmed that there are direct projections from the CeA to the RVLM, and the excitability of these projection neurons can be regulated by acetic acid (a metabolite of ethanol) *via* NMDA receptor activation [58].

#### Rostral Ventrolateral Medulla

RVLM acts as a critical integration station for the transmission of sympathetic signals to the periphery. When the activity of RVLM neurons is enhanced, signals can be transmitted along the pathways to the IML at all levels of the spinal cord, thereby activating peripheral sympathetic nerves innervating the heart, small arteries, and kidneys. These nerves cooperatively regulate the functions of target organs to elevate blood pressure, ultimately completing the conversion of central sympathetic signals into peripheral effects [59].

### Parasympathetic Nervous System: Central Nuclei, Pathways, and Peripheral Effector Innervation

The functional regulation of distinct peripheral target organs by the parasympathetic nervous system (PNS) relies on craniosacrally derived neural circuits (a hallmark anatomical trait distinguishing it from the sympathetic system’s thoracolumbar pathways) that link specialized central nuclei [e.g., dorsal motor nucleus of the vagus (DMV), nucleus ambiguus (NA)] to effector tissues (Fig. 2). In contrast to the wide-distribution and -innervation of SNS, the PNS exhibits target-restricted synaptic connectivity. For example, NA neurons directly innervate cardiac ganglia to fine-tune heart rate, while DMV neurons selectively project to gastrointestinal mucosa to coordinate digestive processes. Thus far, studies have characterized PNS-specific molecular mechanisms (e.g., tissue-selective cholinergic receptor subtypes) and physiological roles (e.g., post-prandial visceral homeostasis) that underpin its unique regulatory functions.

#### Nucleus Ambiguus

In terms of cardiac regulation, studies have revealed that there are six molecular subtypes of neurons in the NA of adult mice, among which *Npy2r*^+^ nucleus ambiguus neurons represent the key subtype for cardiac vagal regulation. Anatomically, these neurons directly innervate cardiac ganglia. Functionally, their activation can significantly decrease the heart rate *via* the muscarinic signaling pathway and undergo specific activation during the diving reflex [60]. Recent viral tract-tracing techniques have enabled comprehensive brain-wide mapping of the afferent and efferent neural networks directly linked to cardiac regulation, further defining the anatomical basis of central control over cardiovascular function [61]. This finding provides a clear direction for clarifying the molecular mechanisms underlying parasympathetic regulation of heart rate and identifying precise intervention targets.

#### Dorsal Motor Nucleus of the Vagus

In studies on the anti-inflammatory mechanisms of the pancreas, cholinergic neurons in the DMV in the brainstem serve as the central source of pancreatic anti-inflammatory signals. These neurons transmit signals to the pancreas *via* vagal efferent fibers, activate nicotinic acetylcholine receptors (nAChR) in pancreatic tissues, thereby inhibiting pancreatic inflammatory responses and alleviating tissue damage [62]. This pathway provides a novel potential target for the treatment of acute pancreatitis

In studies related to gastric motility regulation, the mechanism by which electroacupuncture at the Neiguan acupoint enhances gastric motility has been confirmed to depend on the brainstem vagus-vagus parasympathetic pathway. Specifically, gastric sensory signals are transmitted to the Nucleus Tractus Solitarii (NTS) in the brainstem *via* vagal afferent fibers. After integration in the NTS, signals are relayed to the DMV *via* γ-aminobutyric acid (GABA) and L-glutamate (L-Glu). Electroacupuncture at the Neiguan acupoint can inhibit GABA transmission from the NTS to the DMV, alleviate the inhibitory effect of the NTS on the DMV, enhance the activity of DMV efferent vagal motor fibers, and ultimately transmit signals to the effector stomach to improve gastric motility [63].

In the regulation of hepatic glucose metabolism, studies have found that pro-opiomelanocortin (POMC) neurons in the arcuate nucleus of the hypothalamus (ARC) can project to the liver *via* parasympathetic cholinergic neurons of the DMV. Optogenetic stimulation of this pathway inhibits parasympathetic nerve output, upregulates the expression of gluconeogenic enzymes, and thereby elevates blood glucose levels. Moreover, insulin-induced hypoglycemia activates POMC neurons associated with this pathway, indicating that this neural pathway plays a key regulatory role in the hypoglycemic response [64].

## Core Members of the Calcium-Activated Potassium Channel Family

### Biological Concepts, Subtype Classification and Coding System of SK Channels

SK channels are members of the calcium-activated potassium channel family [65]. Characterized by both voltage independence and calcium-dependent gating properties [66], they play a pivotal role in the regulation of neuronal excitability within the CNS [67, 68].

In terms of subtype classification and coding system, three distinct functional subtypes have been clearly identified in the CNS, all of which are encoded by the *KCNN* gene family [69, 70]. Specifically, SK1 (also known as KCa2.1) is encoded by *KCNN1*, which encodes 543, 580 and 536 amino acids (aa) in humans (NM_002248, chromosome 19p13.1), mice (NM_032397, chromosome 8) and rats (NM_019313, chromosome 16p14), respectively; SK2 (also known as KCa2.2) is encoded by *KCNN*2, with the corresponding amino acid counts being 579, 574 and 580 aa in humans (NM_021614, chromosome 5q22.3), mice (NM_080465, chromosome 18) and rats (NM_019314, chromosome 18q11), respectively; SK3 (also known as KCa2.3) is encoded by *KCNN*3, which encodes 736, 731 and 732 aa in humans (NM_002249, chromosome 1q21.3), mice (NM_080466, chromosome 3) and rats (NM_019315, chromosome 2q34), respectively [69].

### Structural and Biological Characteristics of SK Channels

The main structure of SK channels is a homotetramer assembled from four α-subunits, with each α-subunit containing six transmembrane domains (S1–S6). Specifically, the P-loop (pore loop) between the S5 and S6 transmembrane segments forms a potassium-selective filter, which serves as the structure enabling the channel to achieve specific potassium ion permeability. In addition, the leucine zipper domain in the intracellular region maintains the stability of the homotetramer through hydrophobic interactions, providing an important guarantee for the structural integrity of the channel [69]. In terms of functional domain division of labor, the N-terminal region of the α-subunit is involved in the channel assembly process, while the cytoplasmic C-terminal domain contains a conserved calmodulin-binding domain (CaMBD). Calmodulin (CaM) specifically binds to CaMBD *via* its own N-terminal and C-terminal globular domains to form a Ca^2+^-sensing complex, which serves as the structural basis for SK channels to achieve calcium-dependent gating [71].

The static structural features and functional domain architecture of SK channels form the molecular foundation for their biological behaviors, precise subcellular localization, and dynamic regulation. In pyramidal neurons, the somatodendritic distribution of SK channels is controlled by sustained cyclic adenosine monophosphate-protein kinase A (cAMP-PKA) signaling intensity. Basal PKA activity not only significantly restricts SK channel expression at the somatic membrane, but also dictates their distribution mode as single channels or multichannel clusters within membrane nanodomains, thus modulating channel nanoclustering and somatodendritic patterning [72]. Regarding cytoskeleton-linked trafficking, ɑ-actinin2, an F-actin cross-linking protein, is essential for the functional membrane targeting of SK2 channels. The membrane localization of SK2 channels is dynamically regulated, with main processes including ɑ-actinin2-mediated cytoskeletal anchoring and endocytic recycling through early endosomes. Disruption of this pathway markedly changes the plasma membrane surface expression of SK2 channels [73].

### Physiological Mechanisms Underlying the Role of SK Channels in Action Potential Generation

The synergistic action of the CaMBD-CaM-mediated functional domain is the basis for SK channels to achieve voltage-independent gating. When action potentials trigger the opening of voltage-gated Ca^2+^ channels (VDCCs), local Ca^2+^ influx leads to an increase in intracellular free Ca^2+^ concentration. Ca^2+^ then binds to the permanently associated CaM in the SK channel complex, inducing conformational changes in both CaM and the channel. This subsequently promotes channel pore opening and mediates K^+^ efflux [74]. This process not only enables membrane repolarization but also generates a slow hyperpolarizing current, namely the medium afterhyperpolarization potential (mAHP) [75, 76]. Notably, N-methyl-D-aspartate receptors (NMDARs) act as an additional Mg^2+^-dependent Ca^2+^ source for SK channel activation: NMDAR opening mediates Ca^2+^ influx, which synergizes with VDCC-derived Ca^2+^ to enhance CaM-SK interaction, forming NMDA-SK coupling that amplifies mAHP amplitude [77–79]. The mAHP can significantly prolong the interval between action potential peaks, thereby restricting the high-frequency firing capacity of neurons and mediating spike frequency adaptation (SFA), the phenomenon where neurons gradually decrease their firing frequency under sustained stimulation [80] (Fig. 3). In brief, through the synergistic effect of structural assembly, calcium signal sensing (including NMDA-SK coupling), and functional regulation, SK channels essentially act as negative feedback regulators of neuronal activity, maintaining the stability of neural activity by inhibiting repetitive firing and preventing hyperexcitability.

While the core structure and gating properties of SK channels are highly conserved across neuronal types [69, 71], most detailed mechanistic studies have been conducted in hippocampal, cortical, or heterologous expression systems. Direct experimental validation of these mechanisms in presympathetic PVN neurons and NA cardiac parasympathetic neurons remains limited.

## Physiological Functions and Molecular Mechanisms of SK Channels in the Regulation of Central Nuclei of the Autonomic Nervous System

Under normal physiological conditions, SK channels act as vital biomolecules for the precise regulation of neuronal excitability in the central nuclei of the ANS. They can exert differential regulation on sympathetic and parasympathetic nerve outputs in different nuclei, and their functions exhibit significant nucleus specificity (Table 1).

### Paraventricular Nucleus of the Hypothalamus

As a major upstream regulatory center for sympathetic outflow, the presympathetic PVN neurons contain distinct subpopulations, in which SK channels collectively suppress neuronal excitability through multiple mechanisms, thereby maintaining blood pressure homeostasis and fluid balance. Under normal physiological conditions, hypothalamic PVN neurons from rats exhibit expression of SK1, SK2, and SK3 channel subtypes [50], whose regulatory functions rely on multiple signaling pathways. These channels generate apamin-sensitive currents that underlie the mAHP, which masks the afterdepolarization (ADP). By reducing the rate of subthreshold depolarization and contributing to SFA, SK channels negatively modulate neuronal firing rate and excitability *via* postsynaptic mechanisms [81]. Intact endoplasmic reticulum (ER) Ca^2+^ stores are essential for maintaining normal physiological functions and ion channel activities in neurons [82]. Meanwhile, activation of SK channels is dependent on ER Ca^2+^ stores and requires conditional initiation *via* the Ca^2+^-CaM complex. Through mAHP, SK channels constrain neuronal excitability and preserve the homeostasis of sympathetic outflow. Conversely, depletion of ER Ca^2+^ stores inhibits SK channel function, leading to elevated neuronal excitability and augmented sympathetic activity [83]. Functional studies have validated that SK channels in this neuronal subtype directly inhibit sympathetic activity and modulate cardiovascular function. Bilateral microinjection of SK channel blockers into the PVN elicits dose-dependent increases in visceral/renal sympathetic nerve activity, mean arterial pressure, and heart rate [50].

Furthermore, the regulatory role of SK channels is not restricted to the presympathetic PVN-RVLM neurons. In parvocellular PVN neurons, functional coupling between SK channels and transient receptor potential vanilloid 4 (TRPV4) mediates osmosensation. Hypotonic stimulation or TRPV4 agonists reduce neuronal action potential firing frequency, an effect that is selectively abolished by TRPV4 inhibitors or SK channel antagonists, but not by inhibitors of intermediate-conductance (IK) or large-conductance (BK) Ca^2+^-activated K^+^ channels [84].

Under physiological conditions, in spinally projecting presympathetic PVN neurons in Wistar-Kyoto (WKY) rats, SK channels assemble into a functional molecular complex with CaM and casein kinase 2 (CK2). The low level of CK2-mediated CaM phosphorylation preserves the full physiological activity of SK channels. By generating apamin-sensitive currents and giving rise to the mAHP, these channels suppress neuronal firing frequency and negatively regulate excitability, thereby sustaining sympathetic and cardiovascular homeostasis [85]. In contrast, SK channels in rat PVN-RVLM projecting neurons mediate Ca^2+^-dependent outward currents and generate the mAHP. These channels antagonize the ADP *via* postsynaptic mechanisms, thereby reducing neuronal firing frequency and dampening cellular excitability. Notably, this channel subtype is not involved in the regulation of SFA and exerts no significant effect on miniature excitatory postsynaptic currents (mEPSCs) [86].

### Nucleus Ambiguus

The nucleus ambiguus (NA) serves as a principal nucleus for cardiac parasympathetic regulation. Within this structure, SK channels stabilize cardiac parasympathetic outflow by tuning the electrophysiological properties of parasympathetic cardiac motor neurons (PCMNs). SK channels modulate action potential (AP) repolarization, afterhyperpolarization (AHP), and firing frequency in PCMNs, without altering SFA, representing a key mechanism underlying the negative regulation of excitability in this neuronal population. The afterhyperpolarization current (*I*_AHP_) mediated by SK channels contributes predominantly to the mAHP, which comprises a critical mechanism for maintaining the electrophysiological homeostasis of PCMNs [87].

### Dorsal Motor Nucleus of the Vagus

SK channels and BK channels exhibit distinct functional specialization in the DMV, and together constitute the ion channel mechanism underlying parasympathetic regulation of visceral functions. Studies on dorsal vagal neurons (DVNs) within the DMV of normal Wistar rats demonstrate that DVNs highly express SK3 homomeric channels under physiological conditions. These channels can be specifically blocked by apamin, scyllatoxin, and other selective antagonists, and exert their effects by mediating the mAHP, thereby constraining spontaneous firing frequency without altering action potential duration. In parallel, BK channels modulate action potential duration *via* contributing to AP repolarization and the generation of the fast afterhyperpolarization (fAHP), yet exert no significant impact on firing frequency [88]. The two channels function in a complementary manner to collectively maintain the homeostatic excitability of DVNs, ensuring normal parasympathetic modulation of visceral functions.

### Nucleus Tractus Solitarii

The NTS acts as a key integrative center for cardiorespiratory reflexes, and SK channels differentially regulate neuronal excitability in distinct reflex pathways. The co-expression of three SK channel subunits, SK1, SK2, and SK3, in NTS neurons has been confirmed at the transcriptional level by highly specific in situ hybridization techniques [89]. Under physiological conditions, these SK channels exert negative regulatory and reflex-specific integrative effects based on calcium-dependent conditional activation, and cooperate with other potassium channels to maintain neuronal excitability. They not only ensure the rhythmic regulation of the baroreflex, but also guarantee the defensive responses of cardiorespiratory reflexes, ultimately achieving homeostatic balance of the cardiovascular and respiratory systems [90].

## Pathological Mechanisms of SK Channel Dysfunction in Autonomic Nervous System

In hypertensive rats and neonatal mice of diabetic mothers (NMDM), SK channel dysfunction causes distinct impairments in central regulation of ANS (Fig. 4).

## SK Channel Dysfunction in Presympathetic PVN Neurons: Pathogenic Mechanisms of Hypertension

As a primary hub for sympathetic regulation, presympathetic PVN neurons play a critical role in the pathological progression of HTN *via* the dysfunction of SK channels in rats.

In rats subjected to long-term high-salt diet intervention, SK channel currents in presympathetic PVN neurons projecting to the RVLM are significantly reduced. These neurons exhibit shortened interspike intervals (ISI), decreased SFA, increased firing frequency, and enhanced excitability [91]. This finding confirms that high salt intake impairs SK channel function to enhance neuronal excitability, which represents an important predisposing factor for sympathetic overactivation in SSH. This mechanism has been further verified in an HTN model induced by angiotensin II (ANG II) combined with a high-salt diet. In PVN-RVLM projecting neurons, the amplitude and density of SK channel currents are decreased, and the mAHP is attenuated or even eliminated. Blocking SK channels in the PVN of normal rats can recapitulate the phenotypes observed in the hypertensive group, including the disappearance of mAHP, the emergence of ADP, and the elevation of neuronal excitability [81]. Notably, the ANG-II-salt model directly drives sympathetic activation and contributes to the pathogenesis of HTN by reducing SK channel currents and disrupting the mAHP [92].

Further mechanistic investigations have revealed that in spontaneously hypertensive rats (SHR), the abnormal activation of CK2 within the PVN reduces the Ca^2+^ sensitivity of calmodulin bound to SK channels *via* phosphorylation, thereby indirectly inhibiting the current activity of SK channels. Targeted inhibition of CK2 can restore channel function and neuronal excitability, as well as ameliorate sympathetic nerve activity abnormalities associated with blood pressure [85]. Meanwhile, long-term high-salt intake impairs the function of the ER calcium store in PVN neurons, disrupts intracellular calcium signal homeostasis, and indirectly enhances neuronal excitability, which supplements the neurogenic mechanism of SSH [83].

## SK Channel Dysfunction in Nucleus Ambiguus Parasympathetic Neurons: Autonomic Disorders in Neonatal Mice of Diabetic Mothers

As a pivotal central node for the autonomic regulation of the heart, the dysfunction of SK channels in PCMNs of the NA constitutes an important central pathological basis for cardiac ANS dysfunction in NMDM. Maternal diabetes mellitus (DM) exerts a significant pathological impact on SK channel function. Studies have shown that maternal DM markedly enhances SK channel currents in cardiac motor neurons of the NA in rats, which is specifically characterized by increased amplitudes of apamin-sensitive transient outward currents and *I*_AHP_. This current enhancement alters the properties of neuronal action potentials, such as widening the action potential half-width and increasing the AHP amplitude, thereby inhibiting neuronal excitability and leading to a reduced firing frequency of neurons in response to current stimulation [93]. Notably, maternal DM also increases the BK channel currents in PCMNs, which is manifested by elevated transient outward currents sensitive to charybdotoxin (CTX) and paxilline. This change further widens the action potential half-width. However, BK channel blockers (CTX and paxilline) exert no significant effect on the difference in excitability inhibition of PCMNs between the control group and the NMDM group. In other words, BK channel dysfunction is not involved in the reduction of PCMN excitability induced by maternal DM [94]. This comparison clearly confirms that SK channel dysfunction is the predominant factor mediating the excitability inhibition of PCMNs in NMDM. Impaired excitability of NA cardiac motor neurons directly affects the vagal regulation of the heart, which may represent an important central mechanism underlying the cardiac ANS dysfunction in NMDM [93, 94].

## Potential Therapies

SK channel dysfunction represents a pathological link in ANS imbalance-related diseases such as HTN and DM. In view of the differential functional abnormalities of SK channels in these two types of diseases, targeted interventions are expected to be implemented through multi-dimensional potential strategies, including epigenetic regulation, post-translational modification regulation, protein-protein interaction regulation, metabolite regulation, as well as regulation by exogenous chemically synthesized agonists and blockers (Table 2). Notably, direct experimental evidence for most of the following regulatory mechanisms remains lacking in the PVN under HTN conditions and the NA under DM conditions. Current evidence is primarily obtained from other brain regions, peripheral tissues, or unrelated disease models; these mechanisms are therefore proposed as hypothetical therapeutic strategies.

### Epigenetic Regulation

Epigenetics regulates the transcriptional or translational processes of SK channel genes, thereby affecting the expression levels of channel proteins, which provides a promising upstream intervention avenue for disease treatment.

#### DNA Methylation

In the CeA of rats with neuropathic pain, DNA methylation can inhibit the transcription of the SK2 gene by increasing the global methylation level of the CpG island in its promoter region and inducing de novo methylation at specific sites such as −765 and −756. These epigenetic modifications impede the binding of transcription factors, including Sp1 and CP1, to the promoter, thereby downregulating the expression of SK2 mRNA and protein (including the SK2-S and SK2-L isoforms) [95]. This mechanism exerts its effects primarily by reducing SK2 channel function, indicating that enhancing DNA methylation may serve as a potential epigenetic strategy to dampen aberrantly elevated SK2 channel activity in the NA, thereby potentially ameliorating vagal inhibition in DM-related neuropathy of ANS.

#### Non-coding RNA Regulation

In human atrial myocytes from patients with atrial fibrillation (AF) and the mouse atrial cardiomyocyte cell line HL-1, miR-499 negatively regulates SK3 channel function by specifically binding to the conserved sequence in the 3’-untranslated region (3’-UTR) of *KCNN3* mRNA and assembling into the microRNA-induced silencing complex (miRISC) together with Argonaute (Ago) proteins. This complex captures and accelerates the degradation of *KCNN3* mRNA, reduces SK3 transcript levels, and inhibits SK3 protein synthesis, ultimately resulting in the downregulation of SK3 channel function [96]. The miR-499-SK3 axis specifically suppresses SK3 channel activity, thus potentially offering an RNA-targeted intervention for DM neuropathy of ANS pending further validation *in vivo*.

### Post-Translational Modification Regulation

Post-translational modifications precisely regulate the membrane localization, calcium sensitivity, or protein stability of SK channels, thus emerging as a potential strategy for intervening in SK channel dysfunction, with their mechanisms of action exhibiting subtype specificity and disease targeting properties.

#### Ubiquitination Modification

In mouse hippocampal CA1 pyramidal neurons and cultured hippocampal neurons, the ubiquitin ligase UBE3A specifically binds to the C-terminal domain of the SK2 channel and catalyzes ubiquitination at its lysine residues K506, K514, and K550. This modification initiates the endocytosis of SK2 channels, thereby reducing the abundance of SK2 channels on the synaptic membrane surface. When UBE3A expression is lost, the ubiquitination level of SK2 channels is significantly decreased, and endocytosis is inhibited, leading to aberrant accumulation of the channel protein on the synaptic membrane [97]. Restoring UBE3A-mediated ubiquitination and degradation of SK2 may offer a viable post-translational strategy to normalize SK2 overabundance in the NA and alleviate pathological neuronal hypoexcitability in DM.

#### Phosphorylation Modification

In mouse hippocampal CA1 pyramidal neurons, protein kinase A (PKA) regulates SK2 channel function *via* targeted phosphorylation. The mechanism involves phosphorylation of the Ser568–570 residues within the C-terminal domain of the SK2 channel. This modification induces conformational changes in the channel, which not only directly promote endocytosis of SK2 channels but also enhance UBE3A-mediated ubiquitination and degradation of SK2. Meanwhile, it inhibits recycling of the channel back to the synaptic membrane. These three effects coordinately reduce the abundance of SK2 channels on the synaptic membrane surface. Under pathological conditions of UBE3A deficiency, PKA activation can reverse the aberrant accumulation of SK2 channels at the synaptic membrane through the aforementioned mechanisms, thereby maintaining homeostatic channel function [98]. Based on these mechanisms, PKA activation-mediated enhancement of SK endocytic degradation may represent a promising potential strategy to alleviate excessive SK accumulation in the NA and mitigate parasympathetic inhibition in DM.

In addition to the subtype-specific regulation of SK2 by PKA, CK2 also modulates SK channel function *via* phosphorylation, exerting its effects predominantly on the SK. In dopaminergic neurons of the mouse ventral tegmental area (VTA), CK2 regulates SK channel function *via* targeted phosphorylation, with a predominant effect on the SK. The mechanism involves the formation of a complex between CK2 and SK channels, followed by specific phosphorylation of threonine 79 (T79) on channel-associated CaM. This modification reduces the binding affinity of CaM for Ca^2+^, impairs the formation and function of the Ca^2+^-CaM complex, and subsequently suppresses the activation and opening of SK channels, resulting in decreased channel-mediated K^+^ efflux [99]. Enhancing CK2-mediated CaM phosphorylation may thus represent a promising potential strategy to attenuate excessive SK activation and ameliorate abnormal neuronal excitability in the NA during DM. Conversely, in SHR rats, aberrant hyperactivation of CK2 within the PVN has been directly experimentally verified to impair the gating and function of SK channels, thereby causing excessive sympathetic vasomotor outflow [85]. Pharmacological inhibition of CK2 using the selective inhibitor 5,6-dichlorobenzimidazole (DRB) effectively restores SK channel activity, reverses neuronal hyperexcitability, and reduces sympathetic overactivity [85], indicating that targeted CK2 inhibition is a mechanism-based therapeutic strategy with robust experimental evidence to rescue SK function and alleviate neurogenic HTN.

### Lipid-Mediated Regulation

Phosphatidylinositol 4,5-bisphosphate (PIP2), a membrane phospholipid signaling molecule, acts synergistically with CaM to regulate SK channel activation. In human SK2 (hSK2) channels heterologously expressed *in vitro* and rabbit ventricular cardiomyocytes, Ca^2+^ binding to CaM drives a conformational shift of the channel to an intermediate state, during which PIP2 binds specifically to the conserved residue R395 in the S6 transmembrane segment to form a salt bridge. This interaction disrupts the intersubunit salt bridge between E398 and R395 of adjacent subunits, increases conformational flexibility of the S6 segment, and dilates the intracellular hydrophobic gate. Collectively, these events promote channel opening and K^+^ efflux [100]. Enhancing the PIP2-SK2 channels interaction stabilizes channel opening, which may represent a potential mechanistic template for rectifying the disrupted Ca^2+^ signaling-K^+^ current coupling in PVN and mitigating sympathetic overexcitation in HTN.

### Protein-Protein Interaction Regulation

In HEK293 cells, H9c2 rat cardiomyocytes, and rat cardiac tissue, Junctophilin-2 (JP2) is a membrane-binding protein that anchors the sarcoplasmic reticulum to transverse tubules. Its N-terminus contains a key functional domain, the membrane occupation and recognition nexus (MORN) domain. This domain specifically interacts with the C-terminus of the SK2 channel and promotes its surface membrane localization by mediating retrograde trafficking of SK2, thereby increasing the density of SK2-mediated Ca^2+^-activated K^+^ current and ultimately upregulating SK2 channel function. Mutations within the MORN domain of JP2 (e.g., S101R, Y141H) or silencing of JP2 expression impair surface membrane targeting and current activity of SK2, resulting in downregulated SK2 channel function [101]. Preserving the physiological JP2-SK2 interaction ensures proper membrane localization and current density in cardiomyocytes; this regulatory paradigm may offer conceptual insights for correcting abnormal excitability and sympathetic hyperactivity in HTN.

### Metabolite Regulation

In human coronary artery endothelial cells (HCAECs) and human atrial coronary arterioles isolated from diabetic and non-diabetic patients undergoing cardiac surgery, pyridine nucleotides, as pivotal endogenous metabolites, modulate SK channel function through direct interaction with the channels. Among them, oxidized pyridine nucleotide (NAD^+^) directly potentiates apamin-sensitive current activity of SK channels and increases channel open probability, thereby upregulating SK channel function. In contrast, reduced pyridine nucleotide (NADH) inhibits this current and decreases open probability, ultimately resulting in downregulated SK channel function [102]. NAD^+^-mediated SK upregulation could theoretically ameliorate hyperexcitability in the PVN in hypertension, whereas NADH-mediated SK downregulation might theoretically alleviate excessive inhibition in the NA in diabetes.

### Regulation by Exogenous Chemically Synthesized Agonists and Blockers

A series of small-molecule synthetic agonists and blockers have been developed as potential targeted regulatory molecules for the nucleus-specific functional abnormalities of SK channels associated with ANS disorders in HTN and DM.

SK channel agonists, including 1-EBIO [103, 104], NS309 [105–107], CyPPA [108], CyPPA derivatives [109], NS13001 [110, 111], and CAD-1883 (rimtuzalcap) [112], enhance SK channel activity, suppress hyperexcitability of PVN neurons, and reduce sympathetic hyperactivation, showing potential regulatory value in preclinical studies of HTN. Notably, CAD-1883, a positive allosteric modulator of SK channels, has entered two phase II clinical trials for the treatment of essential tremor and spinocerebellar ataxia [112].

SK channel blockers, including Apamin [113], Cm39 [114], P01 [115], UCL1684 [116, 117], ICAGEN [118], NS8593 [119]and AP30663 [120–122], inhibit abnormally enhanced SK channel activity in the NA, thereby restoring excitability of cardiac vagal neurons and potentially improving DM-related autonomic dysfunction. Importantly, AP30663, a non-selective SK1-SK3 channel inhibitor, has completed phase II clinical trials for atrial fibrillation [120]. These results demonstrate that AP30663 effectively restores sinus rhythm in patients with recent-onset atrial fibrillation with superior efficacy to placebo, providing clinical evidence for SK channel-targeted intervention of atrial fibrillation [120].

These small-molecule compounds with subtype selectivity and target specificity serve as reliable tools for investigating the regulatory mechanisms of SK channels and provide potential targeted therapeutic strategies for central ANS diseases such as HTN and DM.

## Perspectives

Nevertheless, several critical uncertainties and conflicting findings remain to be addressed within this field. While SK channel dysfunction in the PVN and NA has been closely linked to autonomic imbalance in hypertension and diabetes, regulatory patterns differ substantially across experimental models. In ANG II-salt hypertension, for instance, SK channel currents are diminished without altering total protein expression, whereas in SHR rats, functional deficits arise primarily from CK2-mediated phosphorylation of calmodulin associated with SK channels. Meanwhile, the precise, subtype-specific roles of SK1, SK2, and SK3 in the PVN and NA remain incompletely defined, largely due to the widespread use of non-selective pharmacological tools. Furthermore, the upstream pathological triggers, including oxidative stress, inflammation, and metabolic perturbations, that drive SK channel dysregulation in these disease states remain poorly understood. Because all available evidence is derived from rodent studies, it remains unclear whether these mechanisms are conserved in human autonomic nuclei, representing a major barrier to clinical translation.

The multilayered regulatory mechanisms of SK channels reported to date, including epigenetic regulation, post-translational modifications, protein-protein interactions, modulation by metabolites, and exogenous small molecules, provide a rich pool of potential targets and strategies for the intervention of autonomic disorders associated with HTN and DM. However, their clinical translation faces three major unresolved challenges. Most current SK modulators lack sufficient subtype selectivity, which increases the risk of off-target effects. In parallel, the precision and efficiency of targeted delivery to discrete central nuclei such as the PVN and NA remain limited, restricting nucleus-specific modulation. In addition, there is a lack of rigorous preclinical validation, including systematic assessments of long-term safety, efficacy, and dose-response relationships in physiologically relevant large animal models.

Future research should focus on developing highly subtype-selective SK channel modulators, optimizing targeted delivery systems for central nuclei, and systematically assessing the long-term safety and efficacy in large animal models, thereby facilitating the translation of the aforementioned potential strategies from basic research to precision-targeted therapy for ANS diseases.

## Conclusions

The imbalance in ANS homeostasis serves as the fundamental pathological basis for chronic diseases such as HTN and disorders of ANS in the offspring of diabetic maternal mice, with its root cause lying in the abnormal regulation of neuronal excitability within central nervous nuclei. As a pivotal molecular player in this regulatory process, SK channels mediate the mAHP to form a negative feedback loop, functioning as a key mechanism for maintaining the excitation-inhibition homeostasis of neurons.

Under physiological conditions, SK channels exhibit specific functional expression in major sympathetic nuclei (e.g., PVN) and crucial parasympathetic nuclei (e.g., NA). By constraining neuronal excitability, they precisely maintain the balance of sympathetic-parasympathetic nerve output and ensure the normal regulation of systemic physiological functions by the ANS. Under pathological conditions, SK channel function displays nucleus-specific abnormalities. In HTN models, SK channel function is downregulated in presympathetic PVN neurons, leading to neuronal hyperexcitability and enhanced sympathetic nerve activity, which in turn drives blood pressure elevation. In the NMDM models, SK channel function is upregulated in NA parasympathetic cardiac motor neurons, inducing suppressed neuronal excitability and ultimately resulting in cardiac ANS disorders.

Targeting the aforementioned pathological mechanisms, this review summarizes multiple intervention strategies including epigenetic regulation and post-translational modification regulation, providing specific directions for correcting the imbalance in central autonomic regulation by targeting SK channels. These findings not only deepen the understanding of the molecular mechanisms underlying ANS disorder-related pathological conditions but also establish a translational framework from basic research to clinical translation, offering potential targets and experimental evidence for the development of disease-specific intervention regimens.

## Figures and Tables

**Fig. 1:**
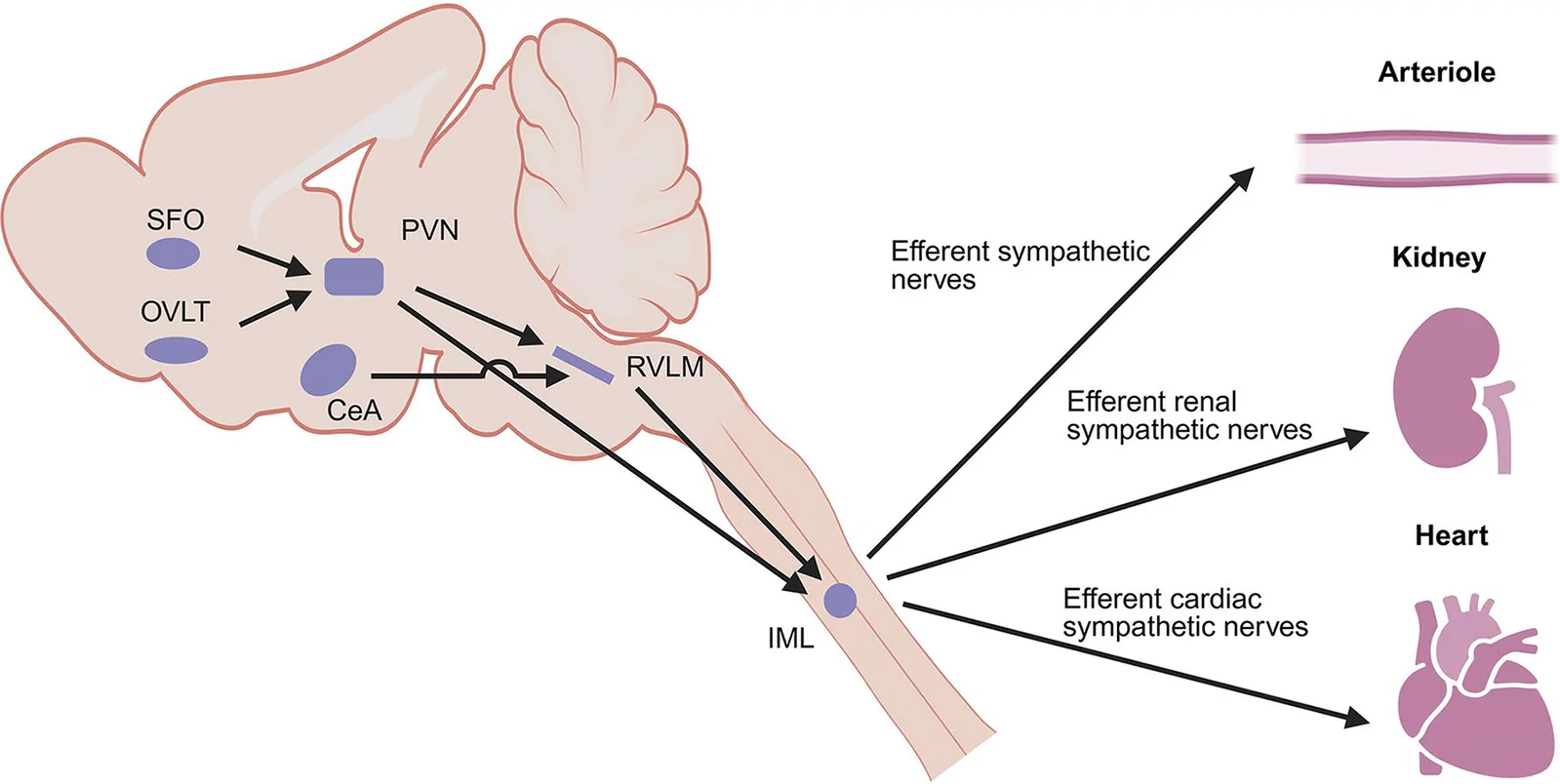
Schematic diagram of the neural regulatory pathway between presympathetic nuclei and peripheral target organs. Central nucleus-specific regions innervate peripheral target organs including small arteries, kidneys, and heart *via* sympathetic outflow. Abbreviations: Subfornical organ, SFO; Organum vasculosum of the lamina terminalis, OVLT; Central nucleus of the amygdala, CeA; Paraventricular nucleus, PVN; Rostral ventrolateral medulla, RVLM; Intermediolateral column of the spinal cord, IML. Created in BioRender. Renjun Wang, Zhengwei Li. (2026) https://BioRender.com/e0gzn4u.

**Fig. 2:**
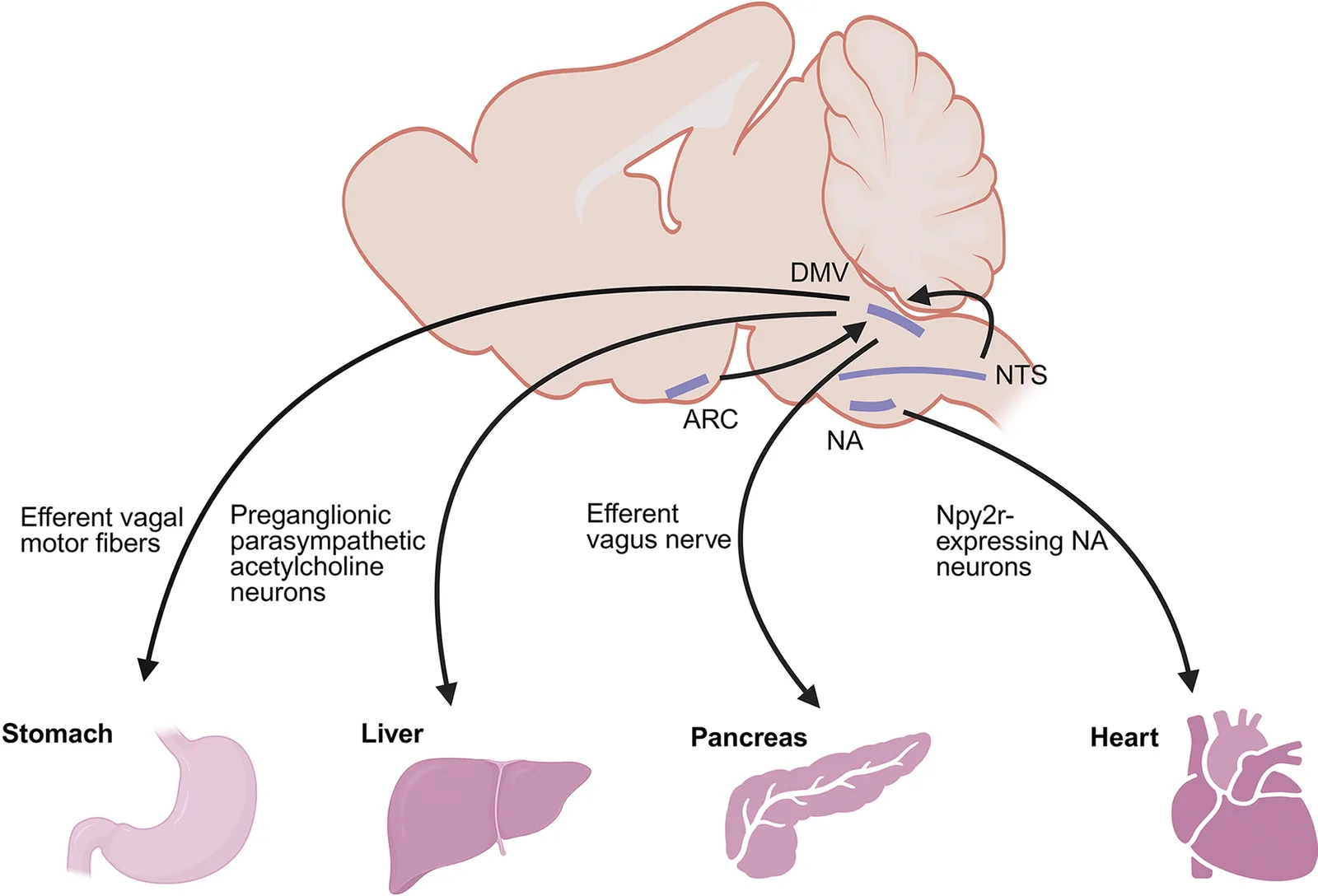
Schematic diagram of the neural regulatory pathway between central parasympathetic nuclei and peripheral effectors. Central nucleus-specific regions innervate peripheral effectors including the heart, stomach, pancreas, and liver *via* efferent fibers of the vagus nerve (parasympathetic nerve). Abbreviations: Nucleus ambiguus, NA; Nucleus tractus solitarii, NTS; Dorsal motor nucleus of the vagus, DMV; Arcuate nucleus of the hypothalamus, ARC. Created in BioRender. Renjun Wang, Zhengwei Li. (2026) https://BioRender.com/d6eur6z.

**Fig. 3:**
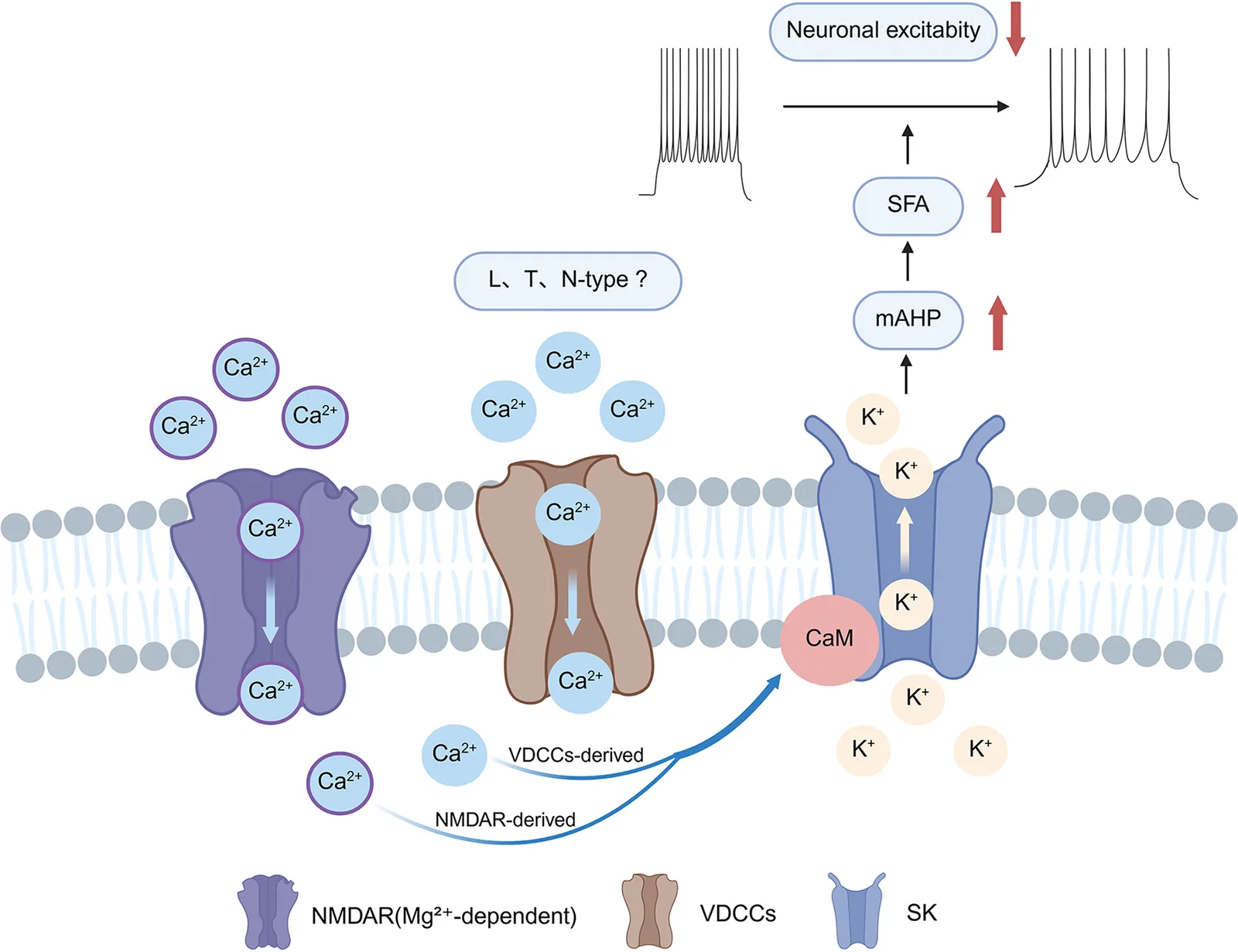
SK channel activation contributes to the generation and amplification of the medium afterhyperpolarization (mAHP) component, thereby regulating neuronal firing frequency. Voltage-gated calcium channels (VDCCs) open upon action potential initiation to mediate Ca^2+^ influx; intracellular Ca^2+^ binds to associated calmodulin (CaM) on SK channels, inducing channel opening and K^+^ efflux to generate mAHP. Meanwhile, neuronal membrane NMDARs open in a Mg^2+^-dependent manner, triggering NMDAR-mediated Ca^2+^ influx. This additional Ca^2+^ source cooperates with VDCC-derived Ca^2+^ to amplify mAHP amplitude, further mediating spike frequency adaptation (SFA) and reducing neuronal excitability. Created in BioRender. Renjun Wang, Zhengwei Li. (2026) https://BioRender.com/w9jggeq.

**Fig. 4:**
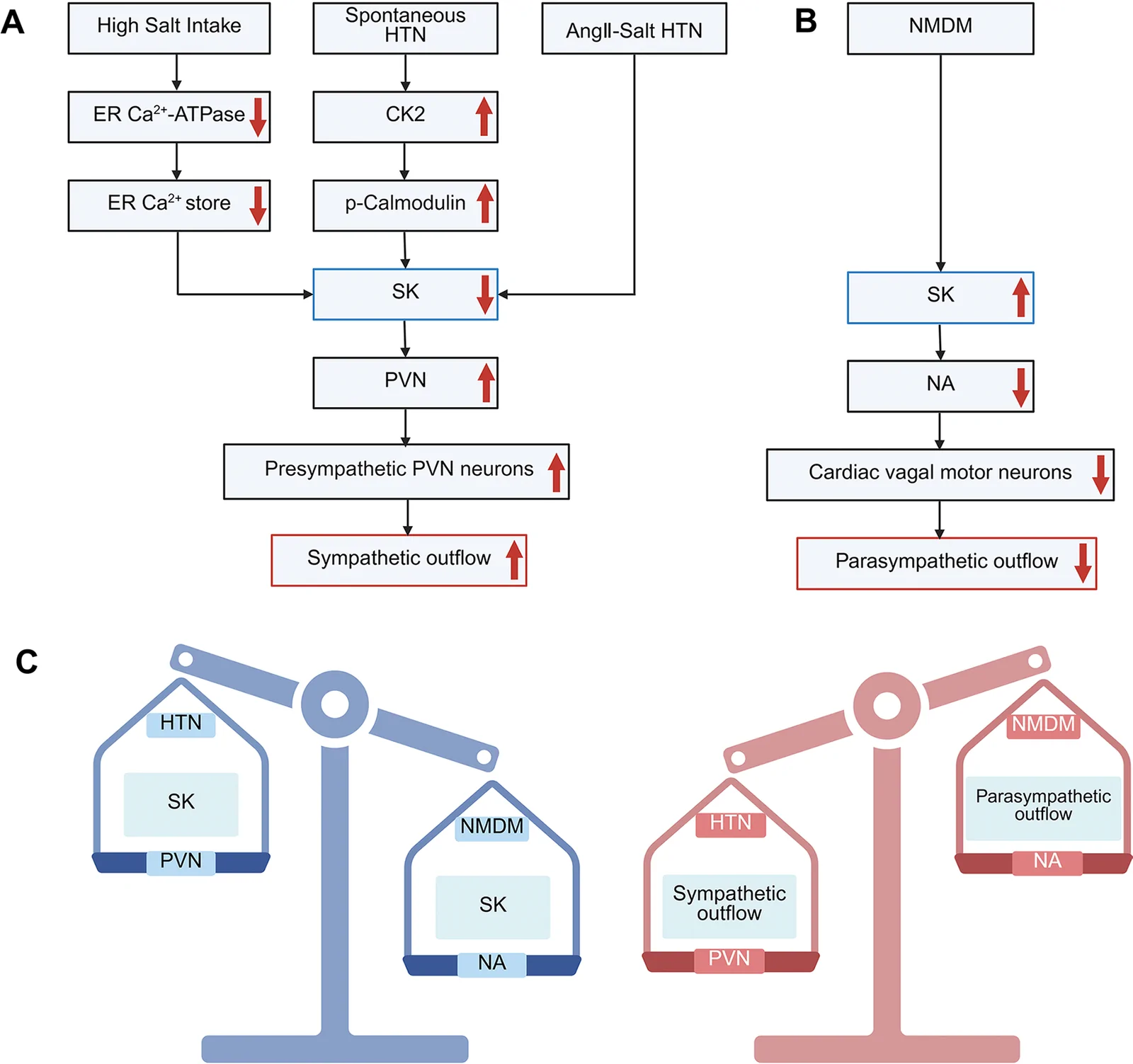
Schematic diagram of nucleus-specific pathological mechanisms of SK channels in autonomic nervous system (ANS) disorders of hypertension (HTN) and neonatal mice of diabetic mothers (NMDM). This schematic diagram depicts a comparison of SK channel dysfunction and the associated autonomic nervous effects in these two chronic diseases. **A** The downregulation of SK channels in the paraventricular nucleus (PVN) of the hypothalamus in different HTN models. Long-term high-salt intake impairs endoplasmic reticulum Ca^2+^-ATPase, disrupts endoplasmic reticulum Ca^2+^ storage, and reduces SK channel activity. Spontaneous hypertension involves the abnormal activation of casein kinase 2 (CK2), which phosphorylates calmodulin (CaM) to inhibit SK channels. In contrast, ANG II-salt HTN directly reduces SK channel currents in the PVN. All of the above processes enhance the excitability of presympathetic PVN neurons and drive excessive sympathetic outflow. **B** The upregulation of SK channels in the nucleus ambiguus (NA) of the offspring of diabetic maternal mice. Maternal diabetes mellitus increases SK channel currents in NA parasympathetic cardiac motor neurons, which reduces neuronal excitability and inhibits parasympathetic nerve output. **C** The nucleus-specific imbalance of SK channels serves as a molecular link connecting SK channel dysfunction to the disorder of autonomic nerve output. Created in BioRender. Renjun Wang, Zhengwei Li. (2026) https://BioRender.com/retsnjj.

**Table 1:** Changes in SK channel function and neuronal excitability of central autonomic neurons under different physiological conditions.

Location	Neuronal type	Condition	SK channel isoform implicated	Signaling event	Channel Response	Neuronal excitability

PVN	Presympathetic PVN neurons	Normal	SK1/SK2/SK3→	Endoplasmic reticulum (ER) Ca^2+^ store and other?	SK channel→	↓ [50, 85]
PVN	Parvocellular PVN Neurons	Hypotonic saline challenge	SK1/SK2/SK3?	Transient receptor potential vanilloid channel (TRPV4) functional coupling	SK channel→	↓ [84]
NTS	NTS neurons	Normal	SK1/SK2/SK3→ [89]	?	SK channel→	↓ [90]
NA	Parasympathetic Cardiac motoneurons (PCMNs)	Normal	SK1/SK2/SK3?	?	SK channel→	↓ [87]
DMV	Dorsal vagal neurones (DVNs)	Normal	SK3→	?	SK channel→	↓ [88]

→: normal expression; ?: uncertain; ↓: decreased neuronal excitability.

**Table 2:** Potential therapeutic strategies for autonomic nervous system(ANS) imbalance-related diseases (e.g., hypertension (HTN), diabetes mellitus(DM)) based on different regulation of SK channel function.

Categories of Regulatory Mechanisms	Specific regulatory methods	Direction of correction	HTN/PVN/ SK channel function or expression	DM/NA/SK channel function or expression	References

Epigenetic regulation	DNA methylation	SK2↓	–	√	[95]
	Delivery of miR-499 mimics	SK3↓	–	√	[96]
Post-translational modification regulation	UBE3A-mediated ubiquitination	SK2↓	–	√	[97]
	PKA-mediated phosphorylation	SK2↓	–	√	[98]
	CK2-mediated phosphorylation	SK3↓	–	√	[99]
Regulation of cellular signal transduction	PIP2 binding regulation	SK2↑	√	–	[100]
Regulation of protein-protein interactions	JP2 regulation	SK2↑	√	–	[101]
Metabolite regulation	Regulation of NAD^+^ levels	SK1/SK2/SK3?↑	√	–	[102]
	Regulation of NADH levels	SK1/SK2/SK3?↓	–	√	[102]
Regulation by exogenous chemically synthesized agonists	1-EBIO	SK1/SK2/SK3↑	√	–	[103, 104]
	NS309	SK1/SK2/SK3↑	√	–	[105–107]
	CyPPA	SK3↑	√	–	[108]
	CyPPA derivatives	SK3↑	√	–	[109]
	NS13001	SK2/SK3↑	√	–	[110, 111]
	CAD-1883	SK2↑	√	–	[112]
Regulation by exogenous chemically synthesized blockers	Apamin	SK2↓	–	√	[113]
	Cm39	SK2↓	–	√	[114]
	P01	SK2↓	–	√	[115]
	UCL1684	SK2/SK3↓	–	√	[116, 117]
	ICAGEN	SK2/SK3↓	–	√	[118]
	NS8593	SK2/SK3↓	–	√	[119]
	AP30663	SK1/SK2/SK3↓	–	√	[120–122]

√: therapeutic relevance; -: no therapeutic relevance; ?: undetermined SK channel subtype; ↑: upregulated SK channel function or expression; ↓: downregulated SK channel function or expression. Abbreviations: Hypertension, HTN; Diabetes mellitus, DM; Ubiquitin-protein ligase E3A, UBE3A; Protein kinase A, PKA; Casein kinase 2, CK2; Phosphatidylinositol 4,5-bisphosphate, PIP2; Junctophilin-2, JP2; Nicotinamide adenine dinucleotide, NAD^+^; Nicotinamide adenine dinucleotide hydrogen, NADH; 1-Ethyl-2-benzimidazolinone, 1-EBIO; 6,7-Dichloro-1H-in-dole-2,3-dione3-oxime, NS309; Cyclohexyl- [2-(3,5-dimethyl-pyrazol-1-yl)]-piperidin-1-yl-methanone, CyPPA; N-(4-(4-fluorophenyl)-2-thiazolyl)-2-(naphthalen-1-yl)acetamide, NS13001; N-(4,4-difluorocyclohexyl)-2-(3-methyl-1H-pyrazol-1-yl)-6-morpholinopyrimidin-4-amine, CAD-1883; 37-amino acid peptide derived from scorpion venom, Cm39; Scorpion venom-derived peptide, P01; 2-Chloro-N- [2-(4-methoxyphenyl)ethyl]-benzamide, UCL1684; N-(4-Aminophenyl)-5-chloro-2-naphthalenesulfonamide, ICAGEN; 6,7-Dichloro-1,2,3,4-tetrahydroisoquino-line-1-carbonylguanidine, NS8593; N-(3,5-Difluorophenyl)-6-fluoro-4-methylquinazolin-2-amine, AP30663.
